# Editorial: Extracorporeal organ support: innovations and challenges in critical care

**DOI:** 10.3389/fmed.2026.1993886

**Published:** 2026-10-13

**Authors:** Pedja Kovacevic, Sasa Dragic, Danica Momcicevic, Biljana Zlojutro

**Affiliations:** 1 Medical Intensive Care Unit, University Clinical Centre of the Republic of Srpska, Banja Luka, Bosnia and Herzegovina; 2 Faculty of Medicine, University of Banja Luka, Banja Luka, Bosnia and Herzegovina

**Keywords:** continuous kidney replacement therapy, extracorporeal membrane oxygenation, extracorporeal organ support, hemoadsorption, precision critical care

## From organ replacement to integrated critical care support

Extracorporeal organ support has evolved from isolated organ-replacement techniques into an increasingly integrated component of contemporary critical care (1, 2). Extracorporeal membrane oxygenation (ECMO), continuous kidney replacement therapy (CKRT), hemoadsorption, pathogen-removal devices, and combined extracorporeal platforms are now used across a broad spectrum of conditions, including severe acute respiratory distress syndrome (ARDS), refractory septic shock, cardiogenic shock, cardiac arrest, hyperinflammatory syndromes, poisoning, and multiple organ dysfunction. As technological capability has expanded, so too have unresolved questions concerning patient selection, timing, treatment intensity, monitoring, anticoagulation, complications, pharmacological consequences, resource requirements, and meaningful long-term outcomes (1–3).

The 27 contributions assembled in this Research Topic reflect a transition from technology-centered organ support toward patient-centered, physiology-guided, and multimodal critical care. Collectively, they address five interconnected domains: precision phenotyping and prognostication; the application of ECMO across different diseases and clinical trajectories; complications and individualized bedside management; hemoadsorption and pathogen-directed blood purification; and the integration of extracorporeal support with pediatric, renal, metabolic, and recovery-oriented care. Rather than supporting a single uniform approach, the Research Topic emphasizes that the clinical value of any extracorporeal intervention depends on matching the technology to the patient's evolving biological and physiological phenotype (Figure 1).

**Figure 1:**
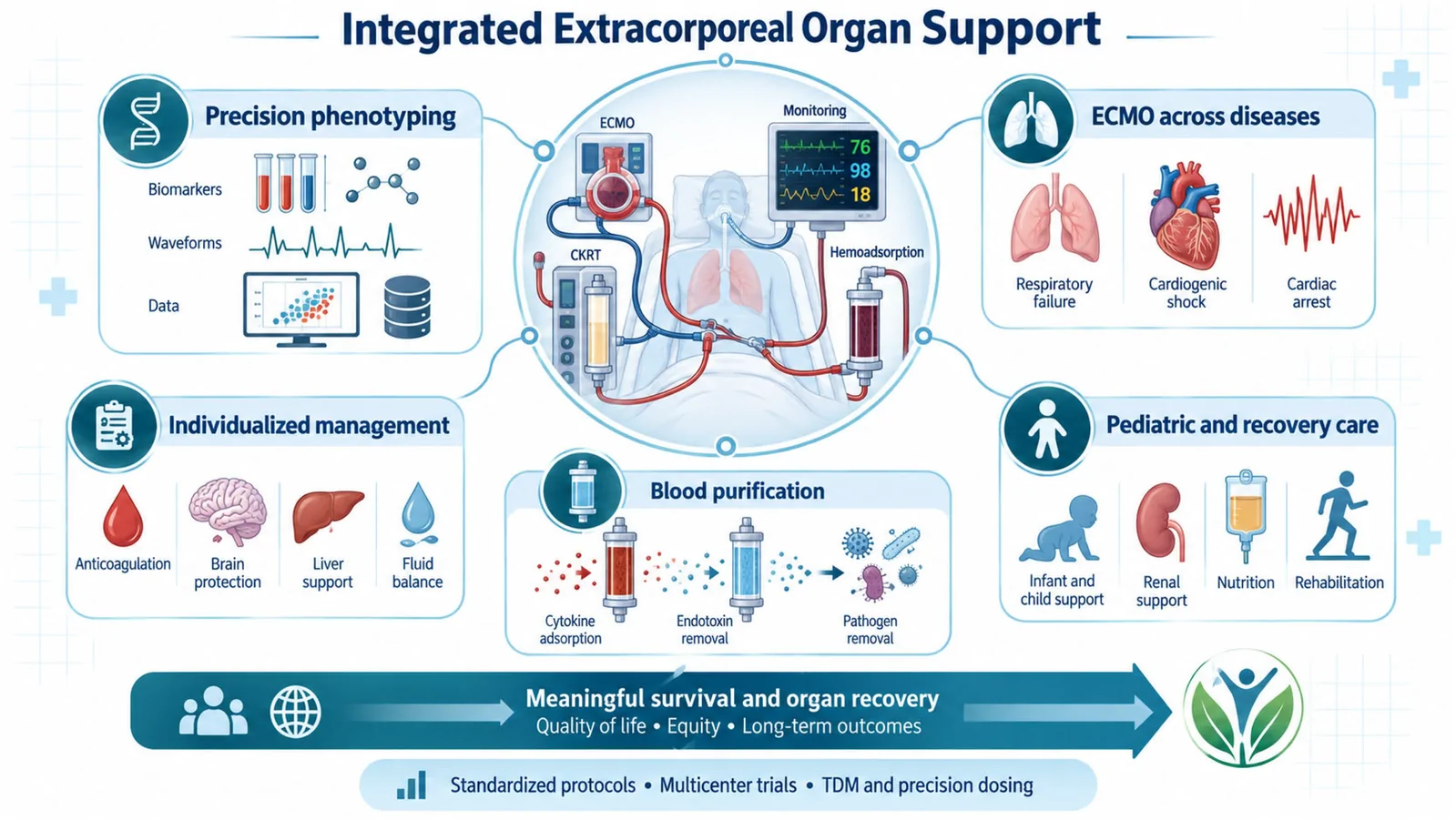
Integrated framework for extracorporeal organ support in critical care. The infographic summarizes the five interconnected domains addressed in this Research Topic: precision phenotyping and prognostication; ECMO across diseases and clinical trajectories; complications and individualized management; hemoadsorption and pathogen-directed blood purification; and pediatric, renal, nutritional, and recovery-oriented care. These domains converge toward the principal goal of extracorporeal support: meaningful survival with organ recovery and acceptable quality of life supported by standardized protocols, multicenter research, therapeutic drug monitoring, and precision dosing.

## Precision phenotyping, monitoring, and prognostication

A central theme of the Research Topic is that patients receiving extracorporeal support are highly heterogeneous. Landoll et al. used high-resolution electronic health record data from 598 adults with severe ARDS receiving VV-ECMO to identify clinically distinct subphenotypes. Differences in inflammation, kidney and liver function, coagulation, and ventilatory variables separated groups with markedly different outcomes, whereas phenotypes based only on ECMO or ventilator settings were less discriminative. These findings suggest that post-cannulation assessment should extend beyond conventional gas-exchange and circuit parameters to include the broader pattern of multiorgan dysfunction.

Several contributions extend this concept through dynamic prognostication. Perez-Garzon et al. evaluated oxygen debt as a mortality marker during VA-ECMO, linking the cumulative burden of impaired oxygen delivery to outcome. Zhang et al. reviewed biomarkers of multiorgan dysfunction on ECMO and considered how persistent lactate elevation, impaired lactate clearance, hyperbilirubinemia, renal dysfunction, and neurological injury may inform recognition of non-recovery and discussions about the goals of care. Importantly, biomarkers should support, rather than replace, multidisciplinary clinical judgment and repeated assessment of reversibility.

The limitations of static scoring systems are considered by Yu et al. in their review of the current SOFA score and the need for its modernization. In a specialized pediatric population, Li et al. assessed pediatric SOFA for outcome prediction among children bridged to heart transplantation with ECMO. Together, these studies support a shift from one-time eligibility decisions toward longitudinal, multidimensional assessment before and throughout extracorporeal support. Future prognostic models will need external validation, transparent calibration, and evaluation of whether their use improves decisions rather than merely predicting adverse outcomes.

## ECMO across diseases and clinical trajectories

The effectiveness of ECMO cannot be separated from the underlying disease (1, 2). Mirus et al. examined the influence of primary pulmonary infection on outcomes in patients with severe ARDS treated with ECMO, highlighting the importance of disease etiology and its interaction with the subsequent course of critical illness. Wang et al. reviewed the pathogenesis of influenza and methicillin-resistant Staphylococcus aureus co-infection, in which epithelial injury, immune dysregulation, and bacterial virulence may produce rapidly progressive necrotizing lung disease. Their analysis reinforces that ECMO can provide physiological support, but survival still depends on prompt identification and effective treatment of the underlying infection.

Rare and complex indications are illustrated by two reports. Tian et al. described ECMO support in severe influenza-associated pulmonary aspergillosis, emphasizing the diagnostic and therapeutic difficulty of invasive fungal disease during profound respiratory failure. Ding et al. reported VV-ECMO for severe exogenous lipoid pneumonia following diesel aspiration. Although individual cases cannot establish effectiveness, they demonstrate how extracorporeal support may create time for diagnosis, source-directed therapy, and recovery in potentially reversible forms of otherwise refractory respiratory failure (1, 2).

Meaningful outcome assessment must also extend beyond decannulation and hospital survival. Ulakcsai et al. reported 24-month outcomes among critically ill COVID-19 patients treated with ECMO, incorporating functional status, psychological recovery, sleep, and quality of life. This long-term perspective is particularly important because survival with severe disability cannot be considered equivalent to recovery. At the opposite end of the clinical trajectory, Yan et al. illustrated the potential of extracorporeal cardiopulmonary resuscitation in unusually prolonged cardiac arrest. The case demonstrates what rapid extracorporeal rescue may achieve in carefully selected circumstances, while also underscoring the need for organized systems, strict selection criteria, and outcome evaluation that includes neurological recovery.

## Complications and individualized management

Complications are not secondary considerations in extracorporeal care; they are major determinants of outcome. In a German registry analysis of 945 COVID-19 VV-ECMO cases, Herrmann et al. evaluated the frequency, distribution, and determinants of bleeding and thromboembolic events. The scale of this cohort gives particular weight to the continuing tension between circuit thrombosis prevention and hemorrhagic harm (3). Complementing this work, Gu et al. compared lower activated clotting time targets during ECMO. Their findings support further individualization of anticoagulation, but retrospective data should not be interpreted as establishing a universal target across different ECMO modes, indications, devices, and bleeding risks (3).

Neurological injury is another major threat. The systematic review and meta-analysis by Dong et al. synthesized risk factors for neurological complications, including stroke and intracranial hemorrhage, and highlights the need for structured neuromonitoring and prevention strategies. Organ-specific complications are further illustrated by Gao et al. through severe hyperbilirubinemia during ECMO in ARDS. This report draws attention to hepatic and biliary dysfunction as potentially important signals of impaired perfusion, hemolysis, congestion, or progressive multiorgan failure.

Bedside management must also remain physiology-driven. Zhou et al. evaluated changes in central venous oxygen saturation and the central venous-to-arterial carbon dioxide tension difference in the assessment of fluid responsiveness. Although not confined to ECMO, this work is relevant to extracorporeal care, where indiscriminate fluid administration can worsen tissue edema and organ function, while inadequate preload may impair native and circuit flow. Taken together, these contributions show that successful extracorporeal support requires simultaneous attention to perfusion, anticoagulation, neurological protection, hepatic function, fluid balance, and circuit integrity rather than a narrow focus on blood flow or gas exchange.

## Hemoadsorption and extracorporeal blood purification

Hemoadsorption represents the second major axis of this Research Topic. Lei et al. reviewed the biological rationale, technical characteristics, and expanding clinical applications of CytoSorb in critical illness. The review also reflects a persistent challenge in this field: plausible mediator removal and improvement in surrogate physiological variables do not necessarily translate into improved survival or functional recovery (4, 5).

The study by Gubensek et al. adds an important procedural dimension by showing that interleukin-6 extraction during prolonged CytoSorb treatment depends on blood flow. This finding suggests that hemoadsorption has a treatment dose determined not only by cartridge selection and duration, but also by circuit configuration and operating conditions. Standardization of these variables will be essential if results are to be compared across studies and translated into reproducible protocols.

Evidence from settings and populations that remain underrepresented in extracorporeal research is provided by two studies from Colombia. Ballesteros et al. examined oXiris-based CKRT in septic shock with acute kidney injury in a resource-limited adult ICU, addressing the feasibility and potential physiological effects of combined renal support, endotoxin adsorption, and cytokine removal. Piñeres-Olave et al. reported a neonatal and pediatric cohort receiving hemoadsorption alongside other extracorporeal support. These reports broaden the geographical and age distribution of the evidence, while also showing why implementation must account for local staffing, infrastructure, cost, vascular access, and monitoring capacity.

Several reports explore hemoadsorption in uncommon hyperinflammatory or toxicological syndromes. Pereira et al. described its adjunctive use in secondary hemophagocytic lymphohistiocytosis, while Castro reported hemoadsorption in Takotsubo cardiomyopathy accompanied by hyperinflammation and multiorgan dysfunction. Hernandez-Vaquero et al. reviewed hemoadsorption as a potential tool in neurotoxic poisoning when conventional treatment is largely supportive and effective antidotes may be unavailable. These contributions are hypothesis-generating: they expand the range of potential indications but cannot determine causal benefit, optimal timing, or appropriate selection criteria.

A broader concept of integrated support is illustrated by Kovacevic et al. who described a physiology-driven, sequential multimodal extracorporeal strategy in refractory septic shock with respiratory, renal, hepatic, and hematological failure. The report is particularly relevant because it moves beyond a single-device paradigm and shows how blood purification and organ-support modalities may be adapted to an evolving clinical phenotype. At the same time, multimodal therapy makes it difficult to attribute improvement to any individual component, reinforcing the need for prospective studies of clearly defined treatment algorithms rather than isolated devices.

The single-center report by Lovric et al. extends blood purification from nonspecific mediator removal toward pathogen-directed treatment using the Seraph 100 filter for ECMO circuit-related infections. This approach is conceptually attractive in patients with persistent infection and a potentially colonized circuit; however, its place relative to source control, antimicrobial therapy, and circuit exchange remains to be defined. Across this group of studies, changes in cytokine concentrations, vasopressor requirements, microbial burden, or other physiological variables should not automatically be equated with clinical effectiveness. Controlled studies with standardized indications, treatment dose, timing, and patient-centered endpoints remain necessary (4, 5).

## Pediatric, neonatal, renal, and recovery-oriented care

The challenges of extracorporeal support are amplified in neonates and infants. Zhauarova et al. reviewed extracorporeal kidney support in this population, including the constraints imposed by extracorporeal circuit volume, vascular access, anticoagulation, hemodynamic instability, and limited device adaptation. Considered alongside the pediatric studies of hemoadsorption and ECMO-bridged transplantation, this review emphasizes that children cannot be managed simply through scaled-down adult protocols (6). Prospective registries, age-specific definitions, and pediatric outcome measures are essential.

Extracorporeal technology can sustain failing organs, but it cannot replace comprehensive critical care. Qinyuan et al. reviewed protein metabolism and nutritional support in critically ill adults, with particular attention to hypercatabolic states such as sepsis and severe burns. Although nutrition is not an extracorporeal modality, it is directly relevant to the recovery phase: preservation of muscle mass, metabolic adaptation, rehabilitation, and functional independence are integral to meaningful survival. This contribution complements the long-term ECMO outcome data and reminds clinicians that organ support should be embedded within a broader recovery-oriented strategy.

## Unresolved questions and future directions

Taken together, the Research Topic demonstrates substantial technological and conceptual progress, but also exposes major gaps. Hemoadsorption evidence remains heterogeneous, with limited randomized data and inconsistent definitions of indication, initiation, dose, duration, and stopping criteria. ECMO studies continue to face confounding by indication and center expertise. Prognostic biomarkers and data-driven phenotypes require external validation and prospective assessment of their effect on decisions. Anticoagulation must be individualized without accepting preventable bleeding or thrombosis, while neurological, hepatic, renal, and functional outcomes should be incorporated systematically into future research. Pediatric and neonatal evidence remains limited, and studies from resource-limited settings are still too scarce to establish equitable implementation strategies.

One gap is especially notable in relation to the original aims of this Research Topic. Although antimicrobial management and pharmacokinetic and pharmacodynamic alterations were identified as priorities, the effects of ECMO, CKRT, and hemoadsorption on antimicrobial exposure remain insufficiently represented in the final collection. Drug sequestration within extracorporeal circuits, changes in volume of distribution, variable extracorporeal clearance, filter age, membrane characteristics, and unintended adsorption may all contribute to subtherapeutic or toxic concentrations (7–9). Prospective pharmacokinetic studies, therapeutic drug monitoring, and model-informed precision dosing should therefore be central to the next phase of extracorporeal critical care research (9, 10).

Future progress will require multicenter collaboration, common data elements, standardized reporting of device and circuit parameters, and outcome sets that extend beyond short-term physiological improvement. Comparative studies should evaluate not only whether a device removes a target molecule, but whether its use changes the patient's clinical trajectory, organ recovery, survival, quality of life, and use of healthcare resources. Such studies should deliberately include children, older adults, patients with complex comorbidity, and centers operating under different resource constraints.

## Conclusion

The contributions to this Research Topic show that extracorporeal organ support is moving toward an era of integrated, personalized, and data-informed critical care. ECMO, kidney support, hemoadsorption, and pathogen-removal technologies can provide essential physiological support and, in selected circumstances, create time for definitive treatment and organ recovery. Technological feasibility, however, should not be confused with established clinical effectiveness. Patient selection, timing, treatment dose, anticoagulation, pharmacological consequences, resource requirements, and long-term outcomes remain incompletely defined. Ultimately, the value of extracorporeal support should be measured not by successful deployment of a device or temporary correction of physiology alone, but by survival with meaningful organ recovery and an acceptable quality of life.
